# Supplementary material for: Land-use history impacts spatial patterns and composition of woody plant species across a 35-hectare temperate forest plot
Source: PeerJ. 2022 Jan 3;10:e12693. doi: 10.7717/peerj.12693 (PMC8734465; doi:10.7717/peerj.12693)
Supplement: Supplemental Information 1 [file peerj-10-12693-s001.docx]

Table S1. Biomass equations of woody species within the HF ForestGEO plot. bm = biomass (kg), dbh = diameter at breast height (cm).

| Species | Equation | Reference |
| --- | --- | --- |
| *Acer pensylvanicum* | bm = (exp(7.227+1.6478*log(dbh/2.54)))/1000 | (Jenkins et al. 2004) |
| *Acer rubrum* | log(bm)=-2.2202 + 2.3922*log(dbh) | (Finzi et al. 2020) |
| *Acer saccharum* | log(bm)=-1.291 + 2.219*log(dbh) | (Finzi et al. 2020) |
| *Acer* unknown | log(bm)=-2.2202 + 2.3922*log(dbh) | (Finzi et al. 2020) |
| *Alnus incana* | bm = (33.722*(dbh^2.712))/1000 | (Connolly 1981) |
| *Amelanchier laevis* | bm = (71.534*(dbh^2.391))/1000 | (Roussopoulos and Loomis 1979) |
| *Aronia melanocarpa* | log(bm) = -2.2118 + 2.4133*log(dbh) | (Chojnacky et al. 2014); hardwoods, Rosaceae |
| *Betula alleghaniensis* | log(bm)=-1.542 + 2.260*log(dbh) | (Finzi et al. 2020) |
| *Betula lenta* | bm = 0.0629*(dbh^2.6606) | (Ter-Mikaelian and Korzukhin 1997) |
| *Betula papyrifera* | log(bm)=-3.082 + 2.683*log(dbh) | (Finzi et al. 2020) |
| *Betula populifolia* | log(bm)=-1.835 + 2.309*log(dbh) | (Finzi et al. 2020) |
| *Betula* spp*.* | bm = 0.0629*(dbh^2.6606) | (Ter-Mikaelian and Korzukhin 1997) |
| *Castanea dentata* | log (bm) = -1.881 + 2.386*log(dbh) | Finzi et al. 2020; equation for red oak |
| *Crataegus* spp*.* | ln(bm)=(3.6834+2.3405*ln(dbh))/1000 | (Dickinson and Zenner 2010) |
| unknown hardwood | log(bm) = -2.48 + 2.4835*log(dbh) | (Jenkins et al. 2004); General hardwood |
| *Fagus grandifolia* | log(bm)=-1.342 + 2.231*log(dbh) | (Finzi et al. 2020) |
| *Frangula alnus* | bm = ((30.971*(dbh^2.764))/1000 | (Grigal and Ohmann 1977) |
| *Fraxinus americana* | log(bm)=-1.381 + 2.208*log(dbh) | (Finzi et al. 2020) |
| *Fraxinus nigra* | bm=0.1634*(dbh^2.3480) | (Ter-Mikaelian and Korzukhin 1997) |
| *Hamamelis virginiana* | bm = (38.111*(dbh^2.900))/1000 | (Smith and Brand 1983) after (Telfer 1969) |
| *Ilex laevigata* | bm = (53.497*(dbh^3.340))/1000 | (Smith and Brand 1983) after (Telfer 1969) |
| *Ilex mucronata* | bm = (31.532*(dbh^2.819))/1000 | (Smith and Brand 1983) after (Telfer 1969) |
| *Juniperus communis* | bm = (59.205*(dbh^2.202))/1000 | (Brown 1976) |
| *Kalmia latifolia* | bm =.2036*(dbh^1.9162) | (Brantley et al. 2016) |
| *Larix laricina* | bm = 0.1265*(dbh^2.2453) | (Ter-Mikaelian and Korzukhin 1997) |
| *Lindera benzoin* | log(bm) = -2.2118 + 2.4133*log(dbh) | Choznacky et al. 2014; equation Laurelaceae |
| *Lyonia ligustrina* | ln(bm)=(3.6685+1.8205*ln(dbh))/1000 | (Dickinson and Zenner 2010) |
| *Nyssa sylvatica* | log(bm) = -2.48 + 2.4835*log(dbh) | (Jenkins et al. 2004); General hardwood |
| *Ostrya virginiana* | log(bm) = -2.48 + 2.4835*log(dbh) | (Jenkins et al. 2004); General hardwood |
| *Picea abies* | log(bm)=-2.621 + 2.456*log(dbh) | (Finzi et al. 2020) |
| *Picea rubens* | log(bm)=-2.621 + 2.456*log(dbh) | (Finzi et al. 2020) |
| *Picea* spp*.* | log(bm)=-2.621 + 2.456*log(dbh) | (Finzi et al. 2020) |
| *Pinus resinosa* | log(bm)=-2.076 + 2.317*log(dbh) | (Finzi et al. 2020) |
| *Pinus strobus* | log(bm)=-3.293 + 2.603*log(dbh) | (Finzi et al. 2020) |
| *Pinus* unknown | log(bm)=-2.076 + 2.317*log(dbh) | (Finzi et al. 2020) |
| *Populus grandidentata* | bm = 0.0785*(dbh^2.4981) | (Ter-Mikaelian and Korzukhin 1997) |
| *Populus tremuloides* | bm = 0.0637*(dbh^2.6087) | (Ter-Mikaelian and Korzukhin 1997) |
| *Prunus pensylvanica* | bm = 0.9758*(dbh^2.1948) | (Young et al. 1980) |
| *Prunus serotina* | bm = 0.0716*(dbh^2.6174) | (Ter-Mikaelian and Korzukhin 1997) |
| *Quercus alba* | log(bm)=-2.520 + 2.590*log(dbh) | (Finzi et al. 2020) |
| *Quercus rubra* | log(bm)=-1.881 + 2.386*log(dbh) | (Finzi et al. 2020) |
| *Quercus velutina* | log(bm)=-2.821 + 2.659*log(dbh) | (Finzi et al. 2020) |
| *Quercus unknown* | log(bm)=-1.881 + 2.386*log(dbh) | (Finzi et al. 2020) |
| *Rhododendron prinophyllum* | ln(bm)=(3.8799+2.3936*ln(dbh))/1000 | (Dickinson and Zenner 2010); *Viburnum* spp. |
| *Salix* species | bm = (60.153*(dbh^2.202))/1000 | (Connolly 1981) |
| *Sambucus racemosa* | ln(bm)=(3.8799+2.3936*ln(dbh))/1000 | (Dickinson and Zenner 2010);*Viburnum* spp. |
| *Sorbus americana* | bm = (44.394*(dbh^3.253))/1000 | (Roussopoulos and Loomis 1979) |
| *Toxicodendron radicans* | bm = (62.134*(dbh^2.460))/1000 | (Roussopoulos and Loomis 1979);avg. shrub |
| *Toxicodendron vernix* | bm = (62.134*(dbh^2.460))/1000 | (Roussopoulos and Loomis 1979);avg. shrub |
| *Tsuga canadensis* | log(bm)=-2.2712 + 2.3444*log(dbh) | (Finzi et al. 2020) |
| *Ulmus americana* | bm = 0.0825*(dbh^2.468) | (Perala and Alban 1994) |
| Unidentified unknown | bm = 0.45*(exp(0.955+2.426*log(dbh/2.54))) | (Wartluft 1977) |
| *Vaccinium corymbosum* | ln(bm)=(3.6685+1.8205*ln(dbh))/1000 | (Dickinson and Zenner 2010) |
| *Viburnum acerfolium* | ln(bm)=(3.8799+2.3936*ln(dbh))/1000 | (Dickinson and Zenner 2010) |
| *Viburnum alnifolium* | bm = (29.615*(dbh^3.243))/1000 | (Smith and Brand 1983) after (Telfer 1969) |
| *Viburnum cassinoides* | ln(bm)=(3.8799+2.3936*ln(dbh))/1000 | (Dickinson and Zenner 2010) |
| *Viburnum dentatum* | ln(bm)=(3.8799+2.3936*ln(dbh))/1000 | (Dickinson and Zenner 2010) |

References

Brantley ST, Schulte ML, Bolstad PV, Miniat CF. 2016. Equations for estimating biomass, foliage area, and sapwood of small trees in the southern appalachians. Forest Science 62:414–421 DOI 10.5849/forsci.15-041.

Brown JK. 1976. Estimating shrub biomass from basal stem diameters. Canadian Journal of Forest Research 6:153–158 DOI 10.1139/x76-019.

Chojnacky DC, Heath LS, Jenkins JC. 2014. Updated generalized biomass equations for North American tree species. *Forestry* 87:129–151 DOI 10.1093/forestry/cpt053.

Connolly BJ. 1981. Shrub biomass–soil relationships in Minnesota Wetlands. St. Paul: University of Minnesota.

Dickinson YL, Zenner EK. 2010. Allometric equations for the aboveground biomass of selected common eastern hardwood understory species. Northern Journal of Applied Forestry 27:160–165 DOI 10.1093/njaf/27.4.160.

Finzi AC, Giasson M-A, Barker Plotkin AA, Aber JD, Boose ER, Davidson EA, Dietze MC, Ellison AM, Frey SD, Goldman E, Keenan TF, Melillo JM, Munger JW, Nadelhoffer KJ, Ollinger SV, Orwig DA, Pederson N, Richardson AD, Savage K, Tang J, Thompson JR, Williams CA, Wofsy SC, Zhou Z, Foster DR. 2020. Carbon budget of the Harvard Forest long-term ecological research site: pattern, process, and response to global change. Ecological Monographs 90(4):e01423 DOI 10.1002/ecm.1423.

Grigal DF, Ohmann LF. 1977. Biomass estimation for some shrubs from northeastern Minnesota. St. Paul: US Department of Agriculture, Forest Service.

Jenkins JC, Chojnacky DC, Heath LS, Birdsey RA. 2004. Comprehensive database of diameter-based biomass regressions for North American tree species. Gen. Tech. Rep. NE-319. Newtown Square, PA: US Department of Agriculture, Forest Service, Northeastern Research Station. 45 p.[1 CD-ROM]. 319.

Perala D, Alban D. 1994. Allometric biomass estimators for aspen-dominated ecosystems in the Upper Great Lakes. Research Paper NC-314. US Department of Agriculture, Forest Service, North Central Research Station, St. Paul, MN.

Roussopoulos PJ, Loomis RM. 1979. Weights and dimensional properties of shrubs and small trees of the Great Lakes conifer forest. Research Paper NC-178. St. Paul, MN: US Dept. of Agriculture, Forest Service, North Central Forest Experiment Station 178.

Smith WB, Brand GJ. 1983. Allometric biomass equations for 98 species of herbs, shrubs, and small trees. Research Note NC-299. St. Paul, MN: US Dept. of Agriculture, Forest Service, North Central Forest Experiment Station 299.

Telfer E. 1969. Weight–diameter relationships for 22 woody plant species. Canadian Journal of Botany 47:1851–1855 DOI 10.1139/b69-271.

Ter-Mikaelian MT, Korzukhin MD. 1997. Biomass equations for sixty-five North American tree species. Forest Ecology and Management 97:1–24 DOI 10.1016/S0378-1127(97)00019-4.

Wartluft JL. 1977. Weights of the small appalachian hardwood trees and components. Res. Pap. NE-366. Upper Darby, PA: US Department of Agriculture, Forest Service, Northeastern Forest Experiment Station. 4p.

Young HE, Ribe JH, Wainwright K. 1980. Weight tables for tree and shrub species in Maine. Life Sciences and Agriculture Experiment Station. Miscellaneous report (USA).
