# Supplementary material for: Land-use history impacts spatial patterns and composition of woody plant species across a 35-hectare temperate forest plot
Source: PeerJ. 2022 Jan 3;10:e12693. doi: 10.7717/peerj.12693 (PMC8734465; doi:10.7717/peerj.12693)
Supplement: Supplemental Information 2 [file peerj-10-12693-s002.docx]

Table S2. List of woody plant species ≥ 1 cm dbh within the HF ForestGEO plot in 2014.

| Scientific name | Common name | Vegetation type | Family |
| --- | --- | --- | --- |
| *Acer pensylvanicum* | striped maple | tree | Sapindaceae |
| *Acer rubrum* | red maple | tree | Sapindaceae |
| *Acer saccharum* | sugar maple | tree | Sapindaceae |
| *Alnus incana* | speckled alder | shrub | Betulaceae |
| *Amelanchier laevis* | smooth shadbush | tree | Rosaceae |
| *Aronia melanocarpa* | black chokeberry | shrub | Rosaceae |
| *Betula alleghaniensis* | yellow birch | tree | Betulaceae |
| *Betula lenta* | black birch | tree | Betulaceae |
| *Betula papyrifera* | paper birch | tree | Betulaceae |
| *Betula populifolia* | grey birch | tree | Betulaceae |
| *Castanea dentata* | American chestnut | tree | Fagaceae |
| *Crataegus spp.* | hawthorn | shrub | Rosaceae |
| *Fagus grandifolia* | American beech | tree | Fagaceae |
| *Frangula alnus* | glossy false buckthorn | shrub | Rhamnaceae |
| *Fraxinus americana* | white ash | tree | Oleaceae |
| *Fraxinus nigra* | black ash | tree | Oleaceae |
| *Hamamelis virginiana* | witch-hazel | shrub | Hamamelidaceae |
| *Ilex laevigata* | smooth winterberry | shrub | Aquafoliaceae |
| *Ilex mucronata* | mountain holly | shrub | Aquafoliaceae |
| *Ilex verticillata* | winterberry | shrub | Aquafoliaceae |
| *Juniperus communis* | common juniper | shrub | Cupressaceae |
| *Kalmia latifolia* | mountain laurel | shrub | Ericaceae |
| *Larix spp.* | larch | tree | Pinaceae |
| *Lindera benzoin* | spicebush | shrub | Lauraceae |
| *Lyonia ligustrina* | maleberry | shrub | Ericaceae |
| *Nyssa sylvatica* | black gum | tree | Cornaceae |
| *Ostrya virginiana* | hop-hornbeam | tree | Betulaceae |
| *Picea abies* | Norway spruce | tree | Pinaceae |
| *Picea rubens* | red spruce | tree | Pinaceae |
| *Pinus resinosa* | red pine | tree | Pinaceae |
| *Pinus strobus* | eastern white pine | tree | Pinaceae |
| *Populus grandidentata* | big-toothed aspen | tree | Salicaceae |
| *Populus tremuloides* | quaking aspen | tree | Salicaceae |
| *Prunus pensylvanica* | pin cherry | tree | Rosaceae |
| *Prunus serotina* | black cherry | tree | Rosaceae |
| *Quercus alba* | white oak | tree | Fagaceae |
| *Quercus rubra* | northern red oak | tree | Fagaceae |
| *Quercus velutina* | black oak | tree | Fagaceae |
| *Rhododendron prinophyllum* | early azalea | shrub | Ericaceae |
| *Salix spp.* | willow species | shrub | Salicaceae |
| *Sambucus racemosa* | red elderberry | shrub | Adoxaceae |
| *Sorbus americana* | American mountain-ash | tree | Rosaceae |
| *Toxicodendron radicans* | poison ivy | liana | Anacardaceae |
| *Toxicodendron vernix* | poison sumac | shrub | Anacardaceae |
| *Tsuga canadensis* | eastern hemlock | tree | Pinaceae |
| *Ulmus americana* | American elm | tree | Ulmaceae |
| *Vaccinium corymbosum* | highbush blueberry | shrub | Ericaceae |
| *Viburnum acerifolium* | maple-leaved viburnum | shrub | Adoxaceae |
| *Viburnum dentatum* | arrowwood | shrub | Adoxaceae |
| *Viburnum lantanoides* | hobblebush | shrub | Adoxaceae |
| *Viburnum nudum* | withe-rod | shrub | Adoxaceae |
